# Polymorphisms at Residue 222 of the Hemagglutinin of Pandemic Influenza A(H1N1)pdm09: Association of Quasi-Species to Morbidity and Mortality in Different Risk Categories

**DOI:** 10.1371/journal.pone.0092789

**Published:** 2014-03-25

**Authors:** Paola Cristina Resende, Fernando C. Motta, Maria de Lourdes A. Oliveira, Tatiana S. Gregianini, Sandra B. Fernandes, Ana Luisa F. Cury, Maria do Carmo D. Rosa, Thiago Moreno L. Souza, Marilda M. Siqueira

**Affiliations:** 1 Laboratório de Vírus Respiratórios e do Sarampo, Instituto Oswaldo Cruz/ FIOCRUZ, Rio de Janeiro, Rio de Janeiro, Brazil; 2 Laboratório Central de Saúde Pública do Estado do Rio de Grande do Sul -Fundação estadual de produção e pesquisa em saúde seção de virologia, Porto Alegre, Rio Grande do Sul, Brazil; 3 Laboratório Central de Saúde Pública do Estado de Santa Catarina, Florianópolis, Santa Catarina, Brazil; 4 Laboratório Central de Saúde Pública do Estado de Minas Gerais/Instituto Octávio Magalhães e Fundação Ezequiel Dias, Belo Horizonte, Minas Gerais, Brazil; 5 Laboratório Central de Saúde Pública do Estado do Paraná, Curitiba, Paraná, Brazil; University of Rochester Medical Center, United States of America

## Abstract

The D222G substitution in the hemagglutinin (HA) gene of the pandemic influenza A(H1N1)pdm09 virus has been identified as a potential virulence marker, because this change allows for virus invasion deeper into the respiratory tract. In this study, we analyzed D, G and N polymorphisms at residue 222 by pyrosequencing (PSQ). We initially analyzed 401 samples from Brazilian patients. These were categorized with respect to clinical conditions due to influenza infection (mild, serious or fatal) and sub-stratified by risky factors. The frequency of mixed population of virus, with more than one polymorphism at residue 222, was significantly higher in serious (10.6%) and fatal (46.7%) influenza cases, whereas those who showed mild influenza infections were all infected by D222 wild-type. Mixtures of quasi-species showed a significant association of mortality, especially for those with risk factors, in special pregnant women. These results not only reinforce the association between D222G substitution and influenza A(H1N1)pdm09-associated morbidity and mortality, but also add the perspective that a worse clinical prognosis is most likely correlated with mixtures of quasi-species at this HA residue. Therefore, quasi-species may have a critical and underestimated role in influenza-related clinical outcomes.

## Introduction

The pandemic influenza A(H1N1)pdm09 virus was responsible for over 18,449 deaths worldwide from 2009 to 2010 [1]. Although increased mortality is generally associated with novel influenza viruses due to the lack of a preexisting immunity in the general population, several groups of patients at higher risk for complications due to the influenza infection were reinforced or identified in 2009, such as pregnant women, obese individuals, immunocompromised patients and, surprisingly, otherwise healthy young adults, among others [2].

In addition to hosts’ predisposed clinical conditions, some specific polymorphisms in the viral genome may enhance its virulence [3]. Circulating influenza A(H1N1)pdm09 viruses from 2009 to the present are virtually similar, and no major antigenic variation has been found in viral hemagglutinin (HA), which is the viral surface glycoprotein responsible for attachment and a major target of the host’s immune response [2]. However, mutations, such as D222G/E/N (H1 numbering without signal peptide), which are located within the HA receptor-binding domain (RBD), have been positively associated with worse clinical outcomes as early as during the 2009 pandemics; being therefore, a potential virulence marker [2], [3]. The D222G mutation increases viral invasion to deeper areas of the respiratory tract because it amplifies viral tropism by allowing viral binding to sialic acid residues with the α2,6- (α2,6) and α2,3-linkages (α2,3) found in the upper and lower respiratory tract, respectively [4], [5], [6].

For RNA viruses, viral pathogenesis and evolution are two related processes because high mutation rates lead to the generation of quasi-species [7]. Although many of these novel variants may not be viable, others may evade hosts’ immune responses and/or be endowed with enhanced virulence [7]. Thus, by studying the heterogeneity of quasi-species at residue 222 of A(H1N1)pdm09 HA and its association with a worse clinical outcome, insights may be made as to what extent the active process of virus replication evolution influences A(H1N1)pdm09-related morbidity and mortality. Therefore, the heterogeneity at residue 222 was analyzed and found to be associated with poor clinical outcomes of the Brazilian population. In our study, we found that A(H1N1)pdm09 mutants at any distribution at residue 222G/D/N are significantly associated with worse clinical conditions due to influenza and to risk factors than the D222G mutation alone. Moreover, we also found the heterogeneity of amino acids at residue 222 in a representative proportion of A(H1N1)pdm09-infected individuals in Brazil.

## Materials and Methods

### Data collection and ethical statement

Our laboratory is the National Reference Laboratory for influenza and Respiratory Diseases for the Brazilian Ministry of Health (MoH) and we also integrate the World Health Organization (WHO) network for Influenza surveillance, as a National Influenza Center (NIC). As part of these surveillance systems, we continuously receive samples of suspected cases for virological surveillance, which includes different laboratory testing, such as subtyping, antigenic and genetic analyses. Samples are collected from individuals with respiratory influenza-like illness or severe acute respiratory infection (SARI), according to WHO and MoH case definitions [8]. Sociodemographic, clinical and epidemiological data are also collected by the epidemiological surveillance teams in the different Brazilian states, which includes basic information, such as gender, age, city/state of case notification and residence and dates of symptoms onset and sample collection. Personal information such as name and address are confidential and only assessed by the head of Laboratory. At the entrance, samples and forms are coded, in such a way that all analyses remain anonymous for all staff (maintaining confidentiality, in accordance to our ethical protocol declared in the scope of our quality system; ISO 15189).

The majority of samples used in this investigation were collected during Influenza A(H1N1)pdm09 pandemics, a global public health emergency. After pandemics, virtually all samples were collected in the scope of the National Influenza Epidemiological Surveillance Program/MoH, as part of a global and federal public health policy for Influenza control and prevention. For the reasons stated above, a formal approval from our Ethical Committee is not required.

### Patient and sample characteristics

We analyzed samples from 401 A(H1N1)pdm09-confirmed patients, as judged using the WHO/CDC RT-PCR protocol [8]. These samples were nasopharyngeal aspirates (NPAs) (n = 392) and lung tissue fragments (n = 9). Nine positive NPAs from 3 immunocompromised patients previously studied by our group and with prolonged viral shedding were also analyzed [9]. The samples were selected from our laboratory database. Clinical data from these patients were classified as follows: 1) fatal cases, individuals who died due to influenza-related complications; 2) serious infections, individuals with severe symptoms of respiratory infection, such as SARI, pneumonia or who underwent hospitalization due to influenza; 3) mild cases, from patients with no reports of any complications cited. Further classification of our patients was also used, based on comorbidities. These include the following categories: chronic pulmonary disorder, subjects with chronic obstructive pulmonary disease (COPD), asthma, chronic lung disease; immunossupression, individuals with HIV, cancer and who underwent transplantation; metabolic disorder, diabetes, obesity and other disorders; cardiovascular disorder included those reporting hypertension group and chronic heart disease; and a group with any comorbidity was also considered. Subjects with incomplete clinical information were excluded from the classification as having mild, serious or fatal outcomes due to influenza infection. Nevertheless, if clinical information was incomplete, but data from risk factor available, such sample was included in the study.

### RNA extraction, RT-PCR and Pyrosequencing (PSQ) assay

Viral RNA was extracted from NPAs using a QIAmp viral RNA mini kit (Qiagen, Hilden, Germany). Lung tissue fragments were macerated using a TissueRuptor (Qiagen), and RNA was extracted using an RNeasy mini kit (Qiagen). The RT-PCR protocol and PSQ assay were performed as described elsewhere [10]. For PSQ assay, customized dispensation order ATGTAT(CAGT)_6_ was conducted in the PyroMark Q96 ID pyrosequencer (Qiagen) [10]. The analyses were performed based on the quality of the pyrograms and algorithm to calculate the proportion of each variant has been described elsewhere [10]. The PSQ protocol may allow for the detection of approximately 5.0% of the quasi-species. To be more conservative, we raised this cutoff by 50%. Thus, viral sub-populations with mixtures above 7.5% were considered in this study.

### Statistical analyses

Descriptive and bivariate analyses (chi-square/Fisher’s exact test for categorical variables and t-test for means) were employed to assess putative associations between variables of interest and outcomes. Multiple logistic regressions were carried out, considering variables of epidemiological relevance and plausibility. Significance was set when *p* value <0.05. Analyses were performed using SPSS for windows, version 19 (SPSS Inc.)

## Results

### Associations among the heterogeneity of amino acids at residue 222 of the HA with epidemiological data and clinical outcomes

We focused in studying samples from the period when changes at 222 residue were more common in the literature, from 2009 to 2011 [11], [12]. We performed PSQ analyses for 401 samples among these 70 samples presented any type of mutation in this residue, 9 (12.8%) were D222G alone and 61 (87.2%) were mixed viral populations. The presence of polymorphisms was significantly higher during the pandemic period (from April 2009 to August 2010), when compared to 2011 (Table 1). The large majority of the samples were collected from June to September/2009 (epidemiological weeks 26 to 35) and from May to August/2011 (epidemiological weeks 22 to 35) (Table 1) – which represents the autumn/winter in Brazil.

**Table 1:** Sociodemographic, clinical and epidemiological characteristics according to Influenza A (H1N1)pdm09 HA 222 polymorphisms.

Variables	N/Total (%)	Polymorphisms at 222 HA residue			p value*
		D (WT)	G	Mixed^1^	
**Collection period**					< 0.001
Pandemic period	301/401 (75.1)	232/301 (77.1)	8/301 (2.2)	61/301 (15.2)	
Post pandemic period	100/401 (24.9)	99/100 (99.0)	1/100 (1.0)	0/100 (0.0)	
**Geographical regions**					< 0.001
South	227/401 (56.6)	197/227 (86.8)	5/227 (2.2)	25/227 (11.0)	
Southeast	150/401 (37.4)	120/150 (80.0)	4/150 (2.7)	26/150 (17.3)	
Northeast	21/401 (5.3)	11/21 (52.4)	0/21 (0.0)	10/21 (47.6)	
North	3/401 (0.7)	3/3 (100.0)	0/3 (0.0)	0/3 (0.0)	
**Gender**					0.605
Female	221/401 (55.1)	179/221 (81.0)	6/221 (2.7)	36/221 (16.3)	
Male	180/401 (44.9)	152/180 (84.4)	3/180 (1.7)	25/180 (13.9)	
**Origin of samples**					0.093
Upper respiratory tract	392/401 (97.8)	326/392 (83.2)	9/392 (14.5)	57/392 (14.5)	
Lower respiratory tract	9/401 (2.2)	5/9 (55.6)	4/9 (44.4)	0/9 (0.0)	
**Clinical condition**					< 0.001
Mild	52/305 (17.0)	52/52 (100.0)	0/52 (0.0)	0/52 (0.0)	
Serious	161/305 (52.8)	140/161 (87.0)	4/161 (2.5)	17/161 (10.6)	
Fatal	92/305 (30.2)	44/92 (47.8)	5/92 (5.4)	43/92 (46.7)	

WT, wild type D222; Pandemic period (EW 17/2009 to 9/2010); Post pandemic period (EW 22/2011 to 35/2011); Serious cases were defined as those who presented SARI or pneumonia and/or demanded hospitalization; EW, epidemiological week. ^1^G222 or mixed populations - regarded as the polymorphisms G/N; G/D; N/D or N/G/D at the 222 HA residue. *Associations were assessed by Pearson Chi-square tests and *p* value was considered significant when <0.05.

Although Brazil is a continental-wide country which crosses the equator line, all our samples were from its Southern Hemisphere portion. Samples from 4 out of the 5 regions of Brazil were studied, which includes the southern (56.6%), southeastern (37.4%), northeastern (5.3%) and northern (0.7%) regions. As most of the samples were collected in the southern and southeastern regions, where influenza A(H1N1)pdm09 activity was more intense [13], naturally the heterogeneity at HA residue 222 from samples of these regions were more frequent, ranging from 11 to 17% (Table 1). Although we found the higher frequency of quasi-species at 222 residue in northeastern region, the sampling of this region is very small, 21 specimens (Table 1). Besides, as our laboratory receives convenience samples, 12 out of these 21 cases were from patients that deceased and, therefore, were more likely to be infected with mutant viruses.

The mean age of the patients was 27.4 (± 18.2) years (ranging from 0 to 88 years old). There was no statistically significant correlation between the emergence of quasi-species at influenza HA 222 residue with patient’s gender (Table 1). Although no statistical significance was observed for the heterogeneity at 222 residue of influenza HA with respect to the origin of the samples, the very small number of specimens analyzed from the lower respiratory tract may have impaired the statistics.

In virtually all cases of mild infection (n = 52), the wild-type (WT) D222 residue was present in the entire viral population (Table 1). Importantly, the frequency of WT D222 residue significantly diminished as influenza A(H1N1)pdm09-related illness severity increased – being observed in 87.0% and 47.8% of the patients with serious and fatal infections (Table 1), respectively. As previously published [14], our data reinforce a statistically significant association between the D222G mutation with serious and fatal outcomes (Table 1). Most importantly, we found a correlation between mixed amino acid residues, a consequence of quasi-species emergence, with both serious infections and fatal outcomes (Table 1). Of note, and as mentioned above, our data are minimally biased by the use of 9 samples from the lower respiratory tract (lung tissues), which are richer in α2,3 and provide an environment more likely to allow the emergence of mutant viruses at the residue 222.

### Impact of quasi-species at residue 222 in individuals with risk factors, especially pregnant women

Although studies on the D222G mutation have associated it with enhanced A(H1N1)pdm09-related morbidity and mortality [2] for the general population, information for risk categories remains scarce. We stratified our data to analyze putative associations between influenza-associated outcomes and heterogeneity at 222 residue in different risk exposure categories. Naturally, the stratification of our cases decreased the number of specimens studied in the different categories. Nevertheless, among individuals with any risk factor, only 16.0% (20/125) of subjects infected with D222 progressed to death whereas 51.7% (15/29) of those infected by 222G or mixed populations deceased (*p*<0.001) (Table 2). Remarkably, similar finding were met among pregnant woman who deceased, where 20.5% and 61.5% had WT D222 and G or mixed infections, respectively (*p*<0.013), in contrast with other risk categories where none significant differences were found. Of note, among the analyzed samples from pregnant woman, 30 individuals were at the second gestation trimester. From the three patients who reported abortions, two had mixtures of N/G/D at residue 222. Next, we looked at the groups of patients with predisposing conditions as a function of the heterogeneity at residue 222, even though all samples sizes were too small (Table 2). Although no statistical significant correlation was found, an important proportion of individuals with chronic cardiovascular, pulmonary, metabolic and renal disorders, immunodepression or under tabagism who died had quasi-species mixtures with respect to 222 residue.

**Table 2:** Distribution of polymorphisms at 222 HA residue in Influenza A(H1N1)pdm09 fatal cases, according to comorbidity or underlying disease.

Risk factors	N fatal/ Total	Polymorphisms at 222 HA residue							*p value* *
		D	G	G/D	N/D	N/G	N/G/D	G or Mixed population	
Any risk factor	35/154	20/125	3/6	1/9	1/2	3/3	7/9	15/29	< 0.001
Pregnancy	15/47	7/34	1/2	3/4	0/0	3/3	1/4	8/13	0.013
Chronic cardiovascular disorder	11/33	7/26	0/0	0/3	1/1	0/0	3/3	4/7	0.186
Chronic pulmonary disorder	2/24	1/22	0/1	0/0	0/0	0/0	1/1	1/2	0.163
Metabolic disorder	11/27	6/19	1/2	0/1	0/1	1/1	3/3	5/8	0.206
Imunodepression	5/33	4/29	0/0	1/3	0/0	0/0	0/1	1/4	0.500
Tabagism	5/20	4/16	1/2	0/1	0/0	0/0	0/1	1/4	1.00
Chronic renal disorder	3/7	2/6	0/0	1/1	0/0	0/0	0/0	1/1	0.429

* Associations were assessed by Pearson Chi-square tests and *p* value was considered significant when <0.05.

### Prolonged viral shedding and mutants at residue 222

Because we have previously studied immunocompromised patients with prolonged A(H1N1)pdm09 shedding [9], we verified if mutant viruses and their sub-populations would emerge during the course of infection in these patients. We analyzed samples from patients with 9, 31 and 56 days of viral shedding. Positive association between prolonged viral replication and quasi-species emergence were not found. In fact, one patient had a mixture of N/G/D in their viral population at the initial sampling; however, the WT D222 residue had been fixed by day 29 after the initial sampling. All other samples had the WT D222 residue in the entire viral population. Despite these observations, associations between prolonged virus shedding and mixed populations at residue 222 may exist [14]; however, the very small number of patients available for this study did not allow us to make strong statistical inferences.

## Discussion

Considering that the D222G polymorphism of the HA gene has been positively associated with disease severity [2], [3] and that the importance of quasi-species has been highlighted in the context of this residue [11], [14], [15], [16], [17], [18], [19], in this study, we investigated whether any diversity of quasi-species at the 222 residue were associated with morbidity and mortality in Brazil, where about 10% of the global deaths by Influenza A(H1N1)pdm09 infections had occurred [13]. In addition, we also explored the role of comorbidities/underlying diseases in this context. Besides D222G, our results reinforce the importance of HA 222 mixed populations in disease severity and progression to death, especially among pregnant women.

It has been suggested that the sustained transmission of viral strains with the complete D222G mutation is virtually nonexistent [20]. Variants containing the G222 residue occur sporadically, and their presence does not contribute to the formation of phylogenetic groups [19]. In this study, mutants with the complete D222G mutation and different mixtures of quasi-species could be detected in specimens from severely ill and deceased patients. Although our data reinforce the association between the D222G mutation and mortality, our results also indicate that such an association is even stronger for mixtures of quasi-species. In addition, mixtures of quasi-species appear to have an important positive correlation with influenza-related severity.

Viral populations with residue D222 interact more efficiently with α2-6, whereas G222 and N222 strains have expanded tropisms, as these viruses also bind α2-3 [4], [5]. Consequently, mutant viruses are also able to cause infection in the lower respiratory tract, potentially increasing the clinical severity of influenza infection [19], [20]. Interestingly, a large number of mixtures containing WT and mutant viruses were found in NPAs. Because these are specimens from the upper respiratory tract, the detection of diverse viral sub-populations and associations between quasi-species with influenza-related severity and fatality indicate the spread of viral replication throughout the respiratory tract. In other words, the emergence of quasi-species at residue 222 might be more dynamic during the course of influenza infection than the relatively stable detection of the completely changed D222G residue. In a lung autopsy sample from patient who died of viral pneumonia, viral sub-populations with 222D/G/N were found [17], as a high prevalence of polymorphisms at residue 222 were found in cases of fatal pneumonia, during the second wave of the A(H1N1)pdm09 pandemic in Mexico [11]. Thus, the combination of N222, G222 and D222 forming viral sub-populations may be critical, such as has been observed in the Americas where D222N variants were associated with mortality [21]. In accordance with these observations, mixed populations were found in 4 from 9 lung tissue samples from deceased subjects here analyzed [14], [19].

Whereas other studies highlight that the most important change for morbidity and mortality in A(H1N1)pdm09 HA is the D222G mutation, we call special attention to the emergence of quasi-species and, thus, to other amino acid residues less well-associated with worse clinical outcomes. These differences may have occurred because we employed PSQ assays, which we believe lead to more precise results. Other reports on the role of D222G mutation in morbidity and mortality context are based in Sanger sequencing method [20], [22], [23], [24], [25], [26], which is able to identify the predominant viral population. The use of PSQ, allowed us to explore the heterogeneity and contribution of other amino acid substitutions (and their combination) in clinical severity of disease, at the quasi-species level. In our study, 17.5% of the whole sample showed infection by HA-222 mutant viruses, from which G or mixed populations (D/G, G/N, D/N, D/G/N) were found in 12.8% and 87.2%, respectively. Those infected by a mutant virus/mixed population were about 10 times more likely to die from Influenza A(H1N1)pdm09 infection than those infected by the WT virus (*p*<0.001). These results suggest that besides D222G, infections with mixed populations may be critical. Differences in the frequencies of quasi-species mixtures at residue 222 found by us and others [10], [11], [14], [15], [17] may be due to methodologies and/or to the characteristics of the samples that were analyzed in different countries, such as the abundance of specimens from the lower respiratory tract. In Brazil, we detected mixtures containing residue N222, which has been found and positively associated with influenza severity in other regions of the Americas [21].

The identification of D222G viruses in mild cases [20], [24] have been used to refute/alleviate the importance of this mutation in disease severity [14], [20]. In contrast to these studies, only WT viruses (D222) were found among mild cases (*p*<0.001), emphasizing the relevant role of HA-222 mutations in the clinical outcome. This was particularly important among pregnant women – a population severely affected by the 2009 pandemic [27]. In this subsample, D222G and/or mixtures of quasi-species were significantly associated with death, suggesting that identification of mutant viruses may be an important proxy of clinical outcome. Increased levels of sialic acid and expression of α2,3 linkage in respiratory tract have been described during pregnancy [28], what could possibly contribute to the selection of mutant viruses. In an experimental study, D222G mutant virus was also more virulent in pregnant mice [29]; however, mixtures of viruses representing heterogeneous compositions of amino acids at residue 222 were not used as a reference. For other exposure categories, composed of subjects with underlying chronic diseases (cardiovascular, renal, metabolic) or immunodepression, none association between clinical outcome and the presence of HA 222 mutations could be established. Maybe due to a lack of statistical power, since each of those strata was composed by a small subsample.

Finally, our work emphasize the emergence of mixtures of quasi-species at residue 222 in A(H1N1)pdm09 HA in Brazil, a country severely impacted by the 2009 pandemics. We not only reinforce the association of the D222G mutation with influenza severity and fatality but also add the perspective that a poor clinical prognosis is more strongly associated with mixtures of quasi-species at this residue. Therefore, heterogeneous quasi-species may have a critical and underestimated role in influenza-related clinical outcomes.
